# 

**Published:** 1904-04-30

**Authors:** 


### NOTICE TO CORRESPONDENTS.

All MSS., Letters, Books for Review, and other matters intended for the Editor should be addressed The Editor, "The Hospital," The Hospital Buildings, 28 & 29 Southampton Street, Strand, W.C.

The Editor cannot undertake to return rejected MS., even when accompanied by stamped directed envelope.

**Notice to Advertisers and Subscribers.**

All Advertisements, Orders for Copies of The Hospital, and Business Communications should be addressed to **THE MANAGER (Not to the Editor),** The Hospital, 28 & 29 Southampton Street, Strand, London, W.C.

The Cost of Subscription, post free, is as follows:—Per week, 3½d.; per quarter, 3s. 9d.; per half-year, 7s. 6d.; per annum, 15s. Foreign and Colonial:—Per annum, 19s. 6d., payable, in all cases, in advance.

**NOTICE.—Vol. XXXV. of "The Hospital" (October 1903 to March 1904), in handsome cloth binding, is now on sale at the office of this Journal, price 10s. 6d. Cases for binding the Numbers contained in this Volume 2s. Index to Vol. XXXV., 3½d., post free.**

**The Monthly part for May will be ready on May 2. Price 1s., by post, 1s. 3d.**

### The Student of Medicine.

### APPOINTMENTS.

James Wilson Adam, M.B., C.M.Glasg., Physician to Aberdeen Dispensary. **F. W. Brookes**, M.R.C.S., L.S.A., District Medical Officer of the Parish of Lambeth. **A. E. Carsberg**, M.B., B.C.Cantab., Certifying Factory Surgeon for the Bruton District, County Somerset. **James M. Cowie**, M.D., M.Ch., D.P.H., Medical Officer of Health and Public Analyst for the County Borough of Burton-upon-Trent. **A. N. Haig**, M.B., Ch.B.Aberd., District Medical Officer of the Skipton Union. **James St. L. Kirwan**, M.B., B.Ch., R.U.I., Resident Medical Superintendent of the Ballinasloe District Lunatic Asylum. **F. G. Larkin**, L.R.C.P.Edin., M.R.C.S.Eng., Medical Officer of the Grove Park Workhouse of the Greenwich Union. **William Lloyd**, F.R.C.S., Surgeon-in-charge of the Nose, Ear, and Throat Department, St. Pancras and Great Northern Dispensary, W.C. **D. R. Moir**, M.A., M.B.Aberd., Visiting Surgeon to the Hull and Sculcoates Dispensary. **A. R. Moore**, L.R.C.P.&S., District Medical Officer of the Towcester Union. **H. M. Page**, M.D Brux., M.R.C.S Eng., D.P.H., Medical Officer of Health for the Borough of Yeovil. **C. H. R. Pentreath**, B.A., M.B., B.C.Cantab., Resident Medical Superintendent of the Government Sanatorium for Consumption, Cambridge, New Zealand. **W. L. Pritchard**, M.B., C.M.Edin., Certifying Factory Surgeon for the Brynmawr District, County Brecon. **A. R. Spencer**, M.B Lond, M.R.C.S., L.R.C P., Resident Medical Officer of the Hospital for Epilepsy and Paralysis, Maida Vale. **E. G. Storrs**, M.R.C.S., L.R.C.P., D.P.H., Medical Officer of Health for the Overton Rural District. **Cecil Wall**, M.A., M.D.Oxon., M.R.C.P.Lond., Honorary Physician to the Poplar Hospital.

### "The Hospital" Institutional Library.

**"Hospitals and Asylums of the World." 4 Vols., with a Portfolio of Plans, complete £12 12s.**

**"Burdett's Hospitals and Charities, 1903." 5s. net.**

**"Burdett's Official Nursing Directory, 1903." 3s. net.**

**"Cottage Hospitals, General, Fever, and Convalescent." 10s. 6d.**

**"Uniform System of Accounts for Hospitals and Public Institutions." New Edition. 4s. net.**

**"Hospital Expenditure: The Commissariat." 2s. 6d.**

**"A Legal Handbook for the Use of Hospital Authorities." 2s. 6d.**

**"The Cottage Hospital Case Book or Register of Patients." 10s. and 12s. 6d.**

**All these are published by the Scientific Press, Ltd., and may be obtained through any bookseller, or direct from the publishers, 28 and 29 Southampton Street, Strand, London, W.C.**

### HANDBOOKS FOR NURSES.

***NOW READY.***

**The Latest and Best Book on Midwifery.**

### A COMPLETE HANDBOOK OF MIDWIFERY.

By J. K. WATSON, M.D.Edin.
Author of "A Handbook for Nurses."

Crown 8vo. about 350 pp., with 143 Illustrations, bound in cloth, **6/-** net; **6/4** post free.

### A HANDBOOK FOR NURSES.

By J. K. WATSON, M.D.Edin.

New and Revised Edition. Crown 8vo. profusely illustrated, **5/-** net; **5/4** post free.

### NURSING: ITS THEORY AND PRACTICE.

**A Complete Text-Book of Medical, Surgical, and Monthly Nursing.**

By PERCY LEWIS, M.D.

New Edition. Crown 8vo. illustrated, cloth, **3/6** post free.

### ELEMENTARY ANATOMY AND SURGERY FOR NURSES.

By WILLIAM McADAM ECCLES, M.S.Lond., M.B.

Crown 8vo. Profusely Illustrated, cloth. **2/6** post free.

### ELEMENTARY PHYSIOLOGY FOR NURSES.

By C. F. MARSHALL, M.D., B.Sc., F.R.C.S.

Crown 8vo. Illustrated, cloth. **2/-** post free.

### PRACTICAL GUIDE TO SURGICAL BANDAGING AND DRESSINGS.

By WILLIAM JOHNSON SMITH, F.R.C.S.

Demy 16mo. Profusely Illustrated (Suitable for apron pocket), cloth. **2/-** post free.

### SURGICAL WARD-WORK AND NURSING.

By ALEXANDER MILES, M.D.Edin., C.M., F.R.C.S.E.

Second Edition, Enlarged and entirely Revised. Demy 8vo. Copiously Illustrated, cloth gilt.
**3/6** net, **3/10** post free.

### HOSPITAL SISTERS AND THEIR DUTIES.

By EVA C. E. LÜCKES, Matron, London Hospital.

Third Edition, Enlarged and Partly Rewritten, crown 8vo. about 200 pp., cloth gilt. **2/6** post free.

### THE EXAMINATION OF THE URINE.

By J. K. WATSON, M.D., M.B., C.M., Author of "A Handbook for Nurses."

Demy 16mo. cloth boards. **1/-** net, **1/1** post free.

COMPLETE CATALOGUE POST FREE ON APPLICATION.

*The above Works can be obtained from all Booksellers, or of*
THE SCIENTIFIC PRESS, LTD., 28 & 29 Southampton Street, Strand, London, W.C.

